# Support opportunities for second victims lessons learned: a qualitative study of the top 20 US News and World Report Honor Roll Hospitals

**DOI:** 10.1186/s12913-021-07315-1

**Published:** 2021-12-11

**Authors:** Ruby Marr, Anupama Goyal, Martha Quinn, Vineet Chopra

**Affiliations:** 1 Division of Hospital Medicine, Michigan Medicine, University of Michigan, MI Ann Arbor, USA; 2 School of Public Health, University of Michigan, MI Ann Arbor, USA; 3 Patient Safety Enhancement Program, University of Michigan and VA Ann Arbor Healthcare System, MI Ann Arbor, USA

**Keywords:** Peer to peer support, Second victim, Second victim programs, Provider safety, Healthcare safety, Healthcare trauma

## Abstract

**Background:**

Second Victim Programs (SVPs) provide support for healthcare providers involved in a near-miss, medical error, or adverse patient outcomes. Little is known about existence and structure of SVPs in top performing US hospitals.

**Methods:**

We performed a prospective study and interviewed individuals representing SVPs from 20 US News and World Report (USNWR) Honor Roll Hospitals. Telephone interviews were recorded, transcribed, and de-identified. To allow identification of both quantitative and qualitative themes that unified or distinguished programs with SVPs from each other, a content analysis approach was used.

**Results:**

Of the Top 20 UNSWR hospitals, nineteen individuals with knowledge of or involvement in SVPs were identified. One individual represented two hospital systems for the same institution. Thirteen representatives agreed to participate, 12 declined, and 5 did not respond. One individual who initially agreed to participate did not attend the interview. Among twelve representatives interviewed, 10 reported establishment of SVPs at their hospitals between 2011 and 2016. Most program representatives reported that participants sought support voluntarily. Four domains were identified in the qualitative analysis: (a) identification of need for Second Victim Program (SVP); (b) challenges to program viability; (c) structural changes following SVP creation, and (d) insights for success. Driving SVP creation was the need support medical providers following a traumatic patient event. Poor physician participation due to the stigma associated with seeking support was commonly reported as a challenge. However, acceptance of the mission of SVPs, growing recognition of the value of the program across hospital departments, and systematic safety enhancements were cited as key advantages. To ensure success, participants suggested training a variety of volunteers and incorporating SVPs within quality improvement processes.

**Conclusions:**

In this convenience sample, programs for healthcare providers that experience psychosocial or emotional trauma from clinical care were uncommon. Variation in structure, performance, and measures of success among SVPs was observed. A systematic approach to evaluating SVPs is needed to help inform institutions of how to best serve their second victims.

**Supplementary Information:**

The online version contains supplementary material available at 10.1186/s12913-021-07315-1.

## Introduction

First debuted in 2000, the phrase “second victim” describes healthcare providers who experience psychological and emotional trauma after being involved in a near-miss, minor or serious medical error, or unanticipated adverse patient outcome [1]. The trauma experienced by second victims has been well described and reported in published literature [2]. After adverse events, providers report guilt, anger, loss of confidence, and poor job satisfaction [1, 3]. In 2007, Waterman et al. surveyed 3171 physicians among whom 81% reported that medical errors increased anxiety about future errors and professional reputation [2]. Additionally, only 10% of physicians felt supported by their healthcare organizations, despite The Joint Commission’s recommendations for prompt support to second victims [2, 4].

In order to improve health and wellbeing for providers who have experienced adverse events in the work place, Second Victim Programs (SVPs) have been developed [5, 6]. Majed et al., developed a novel surgery-specific SVP where 81% of participants reported a positive impact on the department’s, “safety and support culture.” [7] In 2013, Nationwide Children’s Hospital implemented a multidisciplinary support program using the Scott Three-Tiered Interventional Model of Support, with staff reporting improved emotional state and improved return to work metrics [4, 8].

While SVPs have shown efficacy and are getting more attention, little is known about the structure and operations of these programs. For example, some programs provide support via a peer-based model, while others use qualified professionals such as psychiatrists [4–6, 9]. Some programs are exclusively for physicians, while others encompass all healthcare providers. Further information on the current state of existing programs to inform change is necessary. Given gaps in this knowledge, we conducted interviews with a convenience sample of twelve representatives drawn from the 20 top healthcare institutions as rated by the 2017 United States News and World Report (USNWR) to understand current SVPs [10]. We aimed to gather data to understand current structure, operations, successes and challenges of existing SVPs.

## Methods

### Study design and sampling

Given the lack of a detailed understanding of programmatic processes, we performed an exploratory prospective, qualitative study. By using a convenience sample of Top 20 U.S. News and World Report’s Honor Roll Hospitals in 2017, we purposefully targeted “high-performing” hospitals who we understood would be more likely to have successful SVPs and supporting personnel. A ‘point of contact’ defined as an individual with direct knowledge of or involvement in SVPs, was identified at each of the 20 hospitals by searching the hospital’s website function using phrases such as “Second Victim,” “Safety,” “Quality,” and “Risk.” Individuals were also identified based on information gathered from experts in the field. Recruitment occurred before the COVID Pandemic, during the months of July and August 2018. Identified individuals, one representative from each institution, were invited via email to participate in a 30-min telephone interview. Invitations were sent three times, each 1 week apart. Nonresponses to emails were considered as inaccurate point of contacts. As a final effort, we called the hospital operator directly and asked to be connected with a representative of the program for each institution that did not yet have a designated ‘point of contact’.

### Data collection

Interviews, one from each hospital representative, were conducted over the telephone by a member of the research team (RM) between August and November 2018 using a semi-structured interview guide with open-ended questions. We chose to conduct interviews over other modalities (e.g., surveys), to better explore, contextualize and understand individual interviewees’ experience and knowledge of Second Victim Program (SVP) structure, successes, and barriers. All participants provided verbal consent prior to the interview session. The interview guide focused on key domains including: (a) age and reason for establishing an SVP program; (b) format, utilization rates, participants’ roles, and support structure; (c) barriers to growth; and (d) program success and whether the SVP had led to system changes (Interview Guide - Appendix 1). Interviews including verbal consent, were audio recorded, transcribed verbatim, de-identified, and stored on a password protected computer. Interviews lasted an average of 33 min and ranged from 23 min to 42 min. No hospital representative was interviewed more than once, and transcripts were not shared with participants for comments or corrections.

### Data analysis

To allow identification of both quantitative and qualitative themes that unified or distinguished programs with SVPs from each other, including information on participation, accessibility, content, determinants of success and challenges, we used a content analysis approach to analyze interview data.

A sample of transcripts were read by three members of the study team (RM, AG, MQ) to familiarize themselves with the content and establish a preliminary codebook. Preliminary codes, code definitions, and example text were logged into the codebook. Codes were derived from the main interview guide components (e.g., need for program, challenges, etc.). Two members of the research team then individually coded the transcripts, met to compare coding and discuss any discrepancies, and to ensure consistency and saturation of available codes [11]. All prospective qualitative data was entered into NVivo 12, a qualitative data analysis software (QRS International Inc., Burlington, Massachusetts). Code reports were generated and reviewed by three team members to identify themes and check for coding consistency. Participants did not provide feedback on coding.

### Ethical and regulatory oversight

The Institutional Review Board of the University of Michigan Medical School (IRBMED) gave a Notice of Determination of “Not Regulated” Status for the study designated HUM00146457, thereby not requiring informed consent, though each participant in the study was verbally consented for participation in which consent was recorded and transcribed as part of the data collection process. This designation was given because the proposed study fell under the University of Michigan’s policy for research using publicly available data sets (http://hrpp.umich.edu/initiative/datasets.html). Under this policy and in accordance with federal regulations for human subjects’ research (45 CFR Part 46) IRBMED approval is not required as the data cannot be tracked to a human subject. Additionally, IRBMED approval was not required for this project as no identifiable private information about individual members, employees or staff of the organization was collected.

## Results

**Fig. 1:**
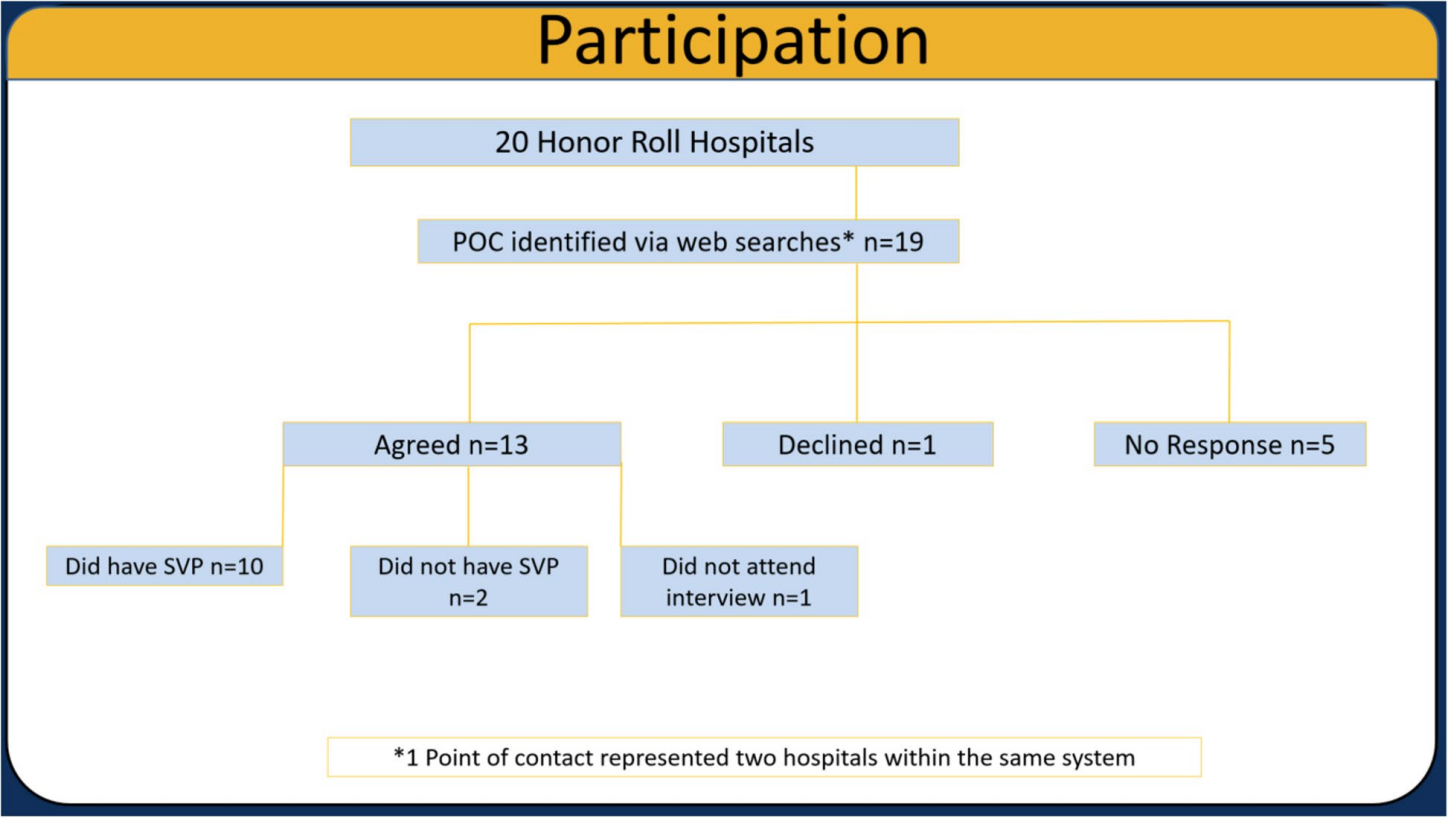
Participation Diagram

Of the Top 20 hospitals, nineteen representatives, one from each institution, were identified via web-searches – one individual represented two hospital systems of the same institution. Of the nineteen representatives, 13 agreed to participate, 1 declined, and 5 did not respond (Fig. 1). One representative who initially agreed to participate did not attend the actual interview. The most often cited reason for non-participation was time constraints and other pressing engagements. Of the twelve representatives interviewed, one from each institution, ten reported that their hospitals had established SVPs. These ten, again one from each institution, were included in the content analysis. Roles of interview participants varied from directors of SVP (*n* = 4), hospital administrators (*n* = 5), to a volunteer (*n* = 1).

Table 1 describes the SVP programs at each of the 10 hospitals involved in this study. Programs were established between 2011 and 2016. The identification of needs for SVP were variable and included adverse patient events (e.g., undetected sepsis resulting in patient death), staff recognition of need, and leadership initiative without an identifiable inciting event. The majority of the programs were open to all employees; with one open to physicians only and one to physicians and advanced practice providers only. Of the 10 programs, six reported tracking utilization rates – a metric that was highly variable. Despite nearly all the SVPs having a formal referral process whereby traumatized medical providers could be referred by peers or referred during quality, safety or risk reviews, most providers sought support on a volunteer basis.

**Table 1:** Structure of the Established Second Victims Programs from Institutions that Participated in the Study

Institution^a^	Inception Year	Identification of need	Access	Participation			Support	
				Program users per unit time	Program utilization was mostly	^b^ Ability to Refer?	Provided by	Mode
A	2011	Adverse event	All employees	20–30/week	Voluntary	No	Trained Peer Volunteers	Face to face
B	2014	Staff Recognition	Anesthesia Department	4–6/month	By Referral	Yes	Trained Peer Volunteers	Face to face
C	2013	Leadership initiative	Physicians and APP’s	Unknown	By Referral	Yes	Trained Peer Volunteers	Phone, email, face to face
D	2013	Adverse event	All employees	Not tracked	Voluntary	Yes	Trained supervisors	Face to face
E	2011	Adverse event	All employees	Unknown	Voluntary	No	Trained Peer Volunteers	Face to face
F	2014	Staff Recognition	All employees	Unknown	Voluntary and by Referral	Yes	Trained Peer Volunteers	Face to face
G	2015	Staff Recognition	Physicians only	1–2/month	Voluntary and by Referral	Yes	Trained Peer Volunteers	Face to face, phone
H	2017	Adverse event	All employees	10/month	By Referral	Yes	Social Worker and Chaplain	Phone, email, face to face
I	2013	Staff Recognition	All employees	18 in 2018	Voluntary	Yes	Trained Elected Peers	Face to face, phone
J	2015	Adverse event	All employees	Unknown	Voluntary	Yes	Pastoral Care Services	Face to face

^a^Institutions assigned a random letter and do not correspond to their USNWR rank

^b^Users could be referred by peers, managers, or from quality, safety and risk reviews

Unknown implies that the interviewee did not have the information

Support sessions were often led by a trained SVP representative which featured peer clinicians (nurses and physicians), but other disciplines including spiritual care providers, unit managers, and social workers were also frequently mentioned as individuals providing support. This support was largely provided in a face-to-face manner. Two programs utilized a staged model, with a second, more intensive tier of support (provided by spiritual care or psychiatrist) offered to those that may need more assistance as determined by SVP staff.

### Key themes related to programs

Several themes from the interviews were identified and organized into four main domains as seen in Table 2: (a) identification of need for program/services; (b) perceived challenges; (c) structural changes following SVP creation; and (d) insights for success. Within the domain of identification of need for program/services, themes such as the recognition of the second victim phenomenon by institutional quality and risk departments, the sequelae of being a second victim, as well as how the risk of being a second victims are heightened when caring for sicker patients were observed. When examining the domain of perceived challenges, we found themes regarding scarcity of resources, lack of awareness of SVP existence, institutional buy-in, barriers to physician participation, barriers in quantifying the number of support encounters, as well as barriers to quantifying programmatic success. Under structural changes following SVP creation, themes emerged around increased institutional recognition and organizational buy-in, learner interest, increased participation, program expansion, as well as enhancements to institutional safety driven SVPs. Within the domain of insights for success, participants suggested ways to establish or expand programs, recruit program users, deliver support, and improve upon an already successful program.

(A) **Identification of Need**

**Table 2:** Common Domains with their Themes from Interviews with Experts

Common Themes	Exemplary Quotes from Interviews
**Domain 1: IDENTIFICATION OF NEED FOR PROGRAM/SERVICES**	
**Recognition by Quality and Risk Departments of need for support for healthcare providers**	*“We were a little worried that we were talking to the people who were involved in RCA, but these weren’t normal events…It was pretty noticeable that we weren’t getting it that everyone may need help or may be struggling.” D page 4.* “And in my role at ***, you know, I oversee all the investigations of all the serious adverse events and the RCAs and just the really bad cases, and I saw the impact it has on providers.” I page 3.
**Sequelae of being a second victim are significant**	*“I mean, they are literally telling you that’s it, ‘I’m quitting’ medicine, I’m quitting surgery, I’m quitting, you know, whatever just because of a bad incident that shook them up so bad.” C page 8.*
**Emotional consequences when providing complex patient care are substantial**	*“She felt at times she was helping the team cope with what was going on as much as the patients and families in the PICU.” H page 4.* *“I had interviewed from EVS staff who worked in the trauma bay and who were quite disturbed when they walked into OR to clean and they were standing in puddles of blood and just felt helpless, you know, like, what had happened here? How am I going to get through this? How am I going to clean this up? How am I going to get through this emotionally?” D page 5.*
**Domain 2: PERCEIVED CHALLENGES TO SVP SUCCESS**	
**Limitation of resources impacts the success of SVPs**	*“It wasn’t a part of my role. I have made it a part of my role, but I have a full time job besides this, so I can’t give it the type of dedication and effort I would like to because I am kind of squeezing it in on my own time...There is no monetary resources for that…it has been something that we do because we think it’s the right thing to do.” B page 7.* *“Now, I say in ‘real time’ because there are two of them right now and depending on how we fund the program moving forward, it may change a little bit. But essentially you know, if they get called in the middle of the night, the idea is, like, do they call back right at that moment? No, they usually call back, like, seven o’clock the next morning.” H page 3.*
**Lack of program awareness and leadership buy-in, impacts SVP success**	*“So, the people that used it, overwhelmingly thought it was effective and it helped them. But the number was small because – the number of people using it was small because of a lack of awareness.” I page 11.* *“The challenges are buy-in from some departments, physician departments and the GM program directors… They have a wellness survey and we tried to incorporate questions as part of their wellness survey. And in the end, we got thrown out.” D page 7.* *“Continuing to educate both the administration about the importance of the program and continuing to receive support.” F page 10.*
**Concern for legal action, confidentiality, and stigma influence physician participation in SVP**	*“They worry that someone is going to find out I had a conversation with this clinician and what if this turns into a legal action and I get subpoenaed?” B page 9.* *“Again the priority is confidentiality, so we can’t go back and ask people involved and so, we discussed it a lot but then decided that confidentiality takes precedence.” G page 7.* *“It is trying to change the culture of like, suffering in silence.” F page 8.* *“Well, I think it’s two things: one, people actually knowing about it and, two, so that getting rid of the stigma about reaching out for help.” I page 4.*
**Lack of systematic tracking effects on sustainability**	*“It’s kind of for them a chicken and an egg situation: they need more funding, but they don’t have any data to kind of back up the work they are doing so it’s a tricky situation.” A page 7.*
**Domain 3: STRUCTURAL CHANGES FOLLOWING SVP CREATION**	
**Increase recognition of SVPs noted at the institution**	“It’s Swartz Rounds, and somebody said, well, was the *** team called? And that was, to me, a measurement of success that somebody was able to say, oh, we have this resource at our hospital and were they called for this adverse event. So, acknowledgement of the program, and they know it’s a resource, they know it’s available.” F page 9.
**Learners are interested in second victim support**	*“They have ramped up direct access to our provider assistance services through after hours support by working with our provider assistance services and creating dedicated target spots just for GME.” H page 10.*
**SVP success results in expansion within the healthcare system**	*“I would say the biggest system change that I have seen is just different clinical areas that didn’t use to ask for help are now asking for help, and one of those examples would be like the emergency department staff.” F page 10.* “It’s really expanded because it’s expanded more into different hospitals in the *** system.” F page 9.
**Increase of buy-in by leadership**	*“We heard a lot of comments around how appreciated the organizational commitment was to putting this program in place, which I think is part of the reason our hospital president this year is like, come on, we have to figure out a way to absolutely keep this going this year.” H page 8.* *“We got some new leadership and I think, in my mind, some pretty forward-thinking people really interested in, you know, safety and quality and just culture…I had a new CMO and a new deputy CMP that were really champions for this…” I page 3.*
**Systematic changes as a result of SVP success**	*“We are retaining more staff members, when people are being more vocal about what they are going through and asking for help.” F page 9.* *“So, for example, if we see like a serious harm event we review as part of those analyses in the event investigation, whether the team needs help and should we deploy those resources to go out and help that team? And we have embedded this all in a policy.” H page 3.*
**Domain 4: INSIGHTS FOR SUCCESS**	
**Increase awareness of SVPs via different educational resources**	*“She continues to give brochures out, to update the brochures. They continue to do the training for staff members. They continue to spread the news, kind of word of mouth. So, there has been a lot of publicity the last couple years, and so it is moving along.” F page 10.* *“I talk about that in orientation with the residents so that they expect it, so it doesn’t feel like people are singling them out. They know that they are going to get contacted if I see a difficult case.” B page 6.*
**Structuring the program in ways that enhance provider interest in seeking support**	*“Physician colleagues talking to physician colleagues. So, rather than having somebody that may be a, you known counselor or psychologist talking to another physician, the thought was when they developed the program was physicians talking to physicians to support them through whatever may have happened.” C page 2.*
**Establish processes to measure SVP impact**	*“We also did a baseline survey before we launched the peer support program, we did a grand rounds presentation about what peer support was and then, we did a survey asking about what has your experience been with difficult cases and adverse events and what helps you? And then, we resurveyed 3 years later to see how people’s perceptions had changed and what was different to try and figure out, were people comfortable adopting this, and, yeah, people seemed to really like the resource.” B page 6.* *“We added 2 questions to our culture of safety surveys, and it was: have you experienced a traumatic event within the last 12 months… What it showed was that people who have an event and were provided support scored higher that those who never had an event and extremely significant increase compared to those who hadn’t received support.” D page 6.*

Interview participants most frequently described that traumatic patient care events led to SVP establishment. They acknowledged that the more complex the patient population, the greater the possibility for error. The trauma experienced by these events had negative impacts on provider wellbeing, productivity, and longevity in their clinical roles.

*“We see lots of bad stuff happen, lots of trauma. We have very difficult, very complex cases that have major risks that don’t go well. People make errors. So, it was seeing the impact on the clinicians we were working with …*” *. B page 2.*

Notably, staff that initiated SVPs often worked in departments such as Risk, Quality, and Patient Safety, and thus directly recognized the importance of support.

*“I do all the quality reviews and in talking with folks who have been involved in, you know, bad cases, inevitably, people would be very upset. You would end up emotionally supporting folks though these terrible events and it felt really wrong to me as the person investigating the case.” B page 2.*

(B) **Perceived Challenges**

Challenges, as perceived by the interviewees, included shortage of resources such as personnel to run the programs and lack of adequate financial support. Frequently interviewees commented on how SVPs lacked sufficient staff or dedicated effort and were doing this in addition to their full-time roles.

*“The main thing I have heard was there is only two of them and to cover a 24/7 pager, they are going to need more staff, even with the volume as is.” H page 8.*

Buy-in from institutional and departmental leadership as well as physician leadership was described as a challenge. Physician participation was reportedly hampered by stigma around asking for support which was a viewed as a ‘sign of weakness.’ What was portrayed was a physician culture of ‘suffering in silence.’ Fear surrounding confidentiality related to medical-legal aspects and possible repercussions from colleagues and supervisors were also cited as limitations to participation.

*“Historically within medicine, you know, there is a huge stigma associated with those people who says ‘I can’t handle it anymore’ or, you know, you are being a wimp. You know you have to buck-up and move on … There is still a stigma associated with asking for help or getting help.” C page 7.*

Lastly, interviewees noted difficulty in collecting data to demonstrate program success. Data collection was hindered by concerns related to confidentiality, legal concerns, and overall burden of this additional work to program volunteers.

*“I tried really hard to gather data from the peer supporters, but people are really busy and are doing this on their own time. There is no reimbursement or compensation for people who do peer support, so I am reluctant to ask them to continue to spend time giving me feedback.” B page 5.*

(III) **Structural Changes following SVP Creation**

Despite described challenges, numerous anecdotes around successful program outcomes were reported. By widening SVP recognition, programs increased participation especially amongst trainees and their program leaders.

*“Over the years … General Medical Education programs have become involved and so have all of their program directors.” D page 1.*

Institutions noted increased staff retention, interest in volunteerism for the program, as well as increased participation over time. SVP expansions occurred across departments and hospitals within health systems with an increase in leadership and organizational commitment.

*“And, over the last several years, people most of the time accept peer support. It’s unusual now for a resident to decline it. And the attending staff, who were the smallest number of people who would accept it, has been steadily growing.” B page 6.*

Likewise, participants reported that program implementation at times led to larger system-level changes, such as incorporation of SVPs within the institution’s quality improvement and event reporting process. One such adjustment involved taking a ‘support time out’ prior to performing additional patient care after experiencing an unanticipated adverse event.

*“So when the patient dies, you know, within 24 hours there is a notification that goes out to the teams. Embedded in that form, there is referral option that says, like, hey, sometimes these things can have a troubling outcome that can lead to [terrible thoughts] this, that, and the other. Here are resources for you to reach out to.” H page 6.*

(IV) **Insights for success**

Tips to promote SVP success spanned several areas. Interviewees suggested that individual programs partner with well-established SVPs and consider launching pilots before expanding to a wider institutional audience.

*“So, that’s why we piloted in those three areas, tried to work out some of the kinks, and then, we went house-wide so, we covered the entire hospital. And since then, for the past year, we have really been working at covering the entire health system.” I page 2.*

In order to increase access to and utilization of SVPs, interviewees recommended disseminating educational resources on the second victim phenomenon and publicizing SVP existence via posters, brochures, flyers and screen savers. To be successful, interviewees stressed the importance of including ‘influencers’ (e.g., managers, providers, leaders etc.) from different units and departments. Influencers have deep understanding of the program, its inherent benefits for participants and its usefulness in daily clinical care. As well, they could serve as champions of the programs making them more visible to staff and faculty who may not be aware of its existence. Many participants suggested creating standardized processes within the institution’s risk, quality, and patient safety reports, to reach medical providers identified at risk of trauma. Some advised performing cost-benefit analyses to garner institutional support.

*“We have trained a lot of people, we offer training to anyone who potentially would like to be a peer supporter, every quarter. And we target unit managers and invite them to be trained, not so they can be care responders, but so they will ultimately know that we exist and refer to us.” A page 7.*

Interviewees described ‘peer-to-peer’ support as the preferred and most successful mode of interaction. As confidentiality is a significant barrier to program participation, SVPs implemented strategies to safeguard provider’s anonymity, as well as protect them from legal repercussions. This was done in a variety of ways such as placing SVPs under the umbrella of Quality Assurance work, which is protected in some states against litigation, to mandating that support conversations be limited to feelings while excluding any details of preceding events which led to the trauma. Some programs developed parameters on ways to connect to a victim (e.g., via telephone rather than email) and format of support provided (e.g., face to face session with focus on second victim feelings instead of adverse event details) to ensure conversations could not be subpoenaed.

*“Yeah, we have a very minimal electronic documentation system that is designed to be essentially not subpoena-able, but has so little information involved that it would not stand up to, you know, a subpoena.” A page 6.*

## Discussion

In this prospective, qualitative study of a convenience sample of top 20 USNWR healthcare institutions, 10 hospitals reported having an established SVP. Most programs were established within the last decade and the most often cited reason for establishment of SVPs was adverse patient events involving providers. SVP structure, access, utilization, format of support, and evaluative measures used across sites also varied considerably. For example, while participation was voluntary at most sites, some institutions relied on a ‘quality or risk review’ referral processes - a finding in keeping with literature that describes the role of risk managers in recognizing and responding to provider fallout from adverse events [12]. Programs were most often accessible to all employees within an institution, however three programs were exclusively for physicians, APPs, or select staff in a department. This variability may be explained by differential recognition of the value of SVPs, with departments rather than institutions recognizing the advantage [13]. Interestingly, even though six of the SVPs had established referral processes in place for second victims, most users sought support voluntarily. Support was predominantly provided by trained peer volunteers in majority of the programs, while two programs used social workers and chaplain. Face to face was the preferred mode of providing support, with some institutions utilizing phone calls and emails in addition.

Qualitative analysis of interview transcripts elucidated four domains including: identification of need, perceived challenges, structural changes following SVP creation, and insights for success. These domains could serve to inform SVP programs in existence, as well as those just getting off the ground about experiences of likely top performing programs. Despite a paucity of quantifiable outcomes, all 10 SVPs interviewed in the study considered themselves successful. Proxies of success included continued recognition and support by institutional leaders, improved scores on culture of safety surveys, and reported positive feedback from participants. Some of the insights for success from programs included launching pilot programs before institutional expansion; use of a variety of media for increasing awareness and promotion of SVPs (e.g., brochures, fliers, talks during resident orientation), use of peer-to-peer support to enhance interest and participation in seeking support from second victims. The most frequently perceived barrier to sustainability was low utilization rates. Physicians in particular were described as unlikely to seek support, potentially due to impressions of perceived weakness, along with concerns of legal repercussions and confidentiality [14, 15]. Attempting to mitigate these factors, SVPs relied on continued education and training to normalize the expected feelings of a second victim, identifying and recruiting second victims to seek support, and leveraging physician-to-physician peer support, thus helping deflect some of the stigma associated with programs [15, 16]. Although ‘time constraints’ to use such programs by physicians have been reported as a barrier to uptake, we did not find this during the interviews [15, 17]. As a result of early success, 5 SVPs were able to implement system-wide changes linking provider well-being to patient safety. Taken together, findings suggest that while SVPs are becoming more common and awareness of these programs is growing, variation in design, approach, integration, and evaluation of such programs remains.

Our study has limitations. First, given the exploratory and qualitative nature of our study design, the sample size was small. We also recruited participants from a convenience sample of hospitals viewed as top performers in clinical care via a national poll; thus, whether findings are generalizable is unknown. Given that SVPs are not yet commonplace, the utilization of top performing hospitals ensured access to information of existent SVPs to perform the review. Second, because interviewees had varying roles within their SVP, the breadth and depth of knowledge about their institutions’ SVP varied, impacting the extent of details garnered in the interviews. However, by using an open-ended approach, we were able to probe for additional gaps and feel confident we learned about all programs. Third, sensitivity to barriers for further promulgation of SVPs may have led to under-reporting of challenges by respondents. Perhaps, an anonymous follow up survey focusing on barriers alone, could overcome this limitation.

Despite these limitations, our study has strengths. To our knowledge, ours is among the first studies to systematically examine SVPs. Substantial variation in program structure and design suggest that a systematic evaluation of these aspects may prove useful. Second, since we used interviews and open-ended questions, we encouraged broad discussions – touching on key aspects such as lessons learned and strategies for new programs that would have been difficult to deduce via surveys. Third, we found that barriers for programs exist and some of these (e.g., stigma) may be addressed relatively easy whereas others (e.g., financial support) may require different approaches. These insights help inform the future planning and propagation of SVPs in hospitals.

In conclusion, in our sample of 10 hospitals, we found that programs for healthcare providers that experience significant psychosocial or emotional trauma from clinical care are not common. Among SVPs in top US hospitals, variation in structure, performance, and measures of success was observed. Since the onset of COVID-19 there has been an increase in healthcare providers’ anxiety, emotional trauma, and self-doubt regarding proficiency and competency following adverse events. Thus, now more than ever, a systematic approach to evaluating and sustaining SVPs is needed to: (a) define and report metrics of success so as to inform other programs of how to best serve their own second victims; (b) establish best practices for SVPs; and (c) evaluate the impact of SVPs on patient-centered outcomes. Further, as most SVPs did not have clear impact or return-on-investment analyses, a dedicated research and policy agenda examining these issues appears necessary.

## Supplementary Information

**Additional file 1.**

## Data Availability

The datasets generated and/or analyzed during the current study are not publicly available to maintain confidentiality of those interviewed as well as their corresponding institutions but are available from the corresponding author on reasonable request. No public data bases were used.

## Abbreviations

SVP: Second Victim Program
SVPs: Second Victim Programs
USNWR: US News and World Report
RM: Ruby Marr
AG: Anu Goyal
MQ: Martha Quinn
