# Supplementary material for: Support opportunities for second victims lessons learned: a qualitative study of the top 20 US News and World Report Honor Roll Hospitals
Source: BMC Health Serv Res. 2021 Dec 11;21:1330. doi: 10.1186/s12913-021-07315-1 (PMC8665707; doi:10.1186/s12913-021-07315-1)
Supplement: Supplementary file 1 — Additional file 1. [file 12913_2021_7315_MOESM1_ESM.docx]

Appendix 1. **Introductory Email and Survey for Institution’s with SVP**

**EMAIL SENT AS INVITATION TO PARTICIPATE:**

Dear Dr. ***,

My name is Ruby Marr, MD and I am a hospitalist at the University of Michigan in Ann Arbor. I am conducting a QI project aiming to learn more about Second Victim Programs. As you may know, a “second victim” is defined as a medical provider who experiences psychological or emotional trauma after being involved in a near miss, medical error, or unanticipated adverse patient outcome. Some institutions have developed these programs for healthcare providers while others have not. I am conducting a survey to better understand the prevalence and design of such programs amongst the top academic institutions in the country. Since yours is one such institution, I am writing to solicit your help for this project.

I would appreciate the opportunity to conduct a 30-minute recorded telephone interview, based on your availability in months July-September. All interviews and information collected will remain completely confidential.

We very much appreciate your participation in this endeavor that may shape resources available to medical providers in the future.

Please do not hesitate to contact me via email should you have any questions. I look forward to hearing from you

Thank you.

Sincerely

Ruby Marr, MD

**SURVEY:**

**Introduction:**

- Interviewer will make introductions.
- Explain the purpose of the interview.
- Guarantee confidentiality from the research team and ask participants to also maintain confidentiality.
- Ask if there are any questions.
- Ask for permission to audio record the interview.

(Note to interviewer: If the participant declines to be recorded, we will ask if we can complete the survey by taking notes of their responses)

*Question 1: Does your Hospital have a support system for medical providers who have experienced a near miss, minor or serious medical error, or unanticipated adverse patient outcome, otherwise known as second victims?

If "yes" skip to question #2

*Question 2: Please tell us the name of your program and the year it was established? Where is your program established?

PROBES:

Medical school?

Certain departments?

Certain hospital units?

*Question 3: Please tell us why the program was established?

PROBES:

Was there an inciting event?

A medical staff request? A request by the office of clinical safety/institution?

Demand for this type of support? Other?

*Question 4: Please tell us who is able to access this program?

PROBES:

RN’s? Techs? Mas? NPs or Pas? Attendings?

Trainees (medical students, interns, residents, fellows)?

Transportation staff? Social work? Case Management?

Any others?

*Questions 5: On average, how many total participants do you have in your program per week/month?

*Question 6: Do you know on average; what percentage of the users are physicians versus non-physician providers?

*Question 7: Please tell us who provides the support?

PROBES:

Colleagues?

Trained professionals dedicated to an individual program?

Outside consultants?

Any others?

*Question 8: How is this support provided?

PROBES:

Individually or group basis?

Is there a prescribed method for support which is structured versus open discussion?

Is it peer to peer or is there a professionally trained support provider?

*Question 9: In what format is this support provided?

PROBES:

Via Internet/technology (for example an electronic application)?

In person/face to face?

Phone? Other?

*Question 10: Could you please tell us if participation is largely:

a) Voluntary vs involuntary

b) Referral based? If yes, who is the referral mostly by (supervisors, co-workers or based on a sentinel committee etc.)

*Question 11: Do you know if Second Victims receive follow-up after an initial encounter? And what percentage?

*Questions 12: Are support sessions documented?

*Questions 13: How do you measure the success of your program?

*Questions 14: Has your program grown/expanded in the past years?

*Question 15: What Challenges does your program face, if any? Please elaborate.

PROBES:

Anonymity?

Aberrant providers?

Participation?

Access?

Advertising?

Funding?

Any other?

*Question 16: How have you addressed these challenges?

*Question 17: Since the establishment of this program, have there been system wide changes to improve patient and provider safety?

a. Could you please give me an example?

Thank you kindly for participating in our telephone interview. We believe that increased awareness of such programs is highly needed and we genuinely appreciate your time and participation.
